# Schistosomiasis control and the health system in P.R. China

**DOI:** 10.1186/2049-9957-1-8

**Published:** 2012-11-01

**Authors:** Charles Collins, Jing Xu, Shenglan Tang

**Affiliations:** 1 Liverpool School of Tropical Medicine, University of Liverpool, Liverpool, UK; 2 National Institute of Parasitic Diseases, Chinese Centre for Disease Control and Prevention, Shanghai, P.R. China; 3 Duke Global Health Institute, Duke University, Durham, NC, 27708, USA

**Keywords:** Schistosomiasis control, Health system, P.R. China

## Abstract

Over the last sixty years advances have been made in the control of schistosomiasis in P.R. China. There are, however, difficult challenges still to be met. This paper looks at the extent to which the health system offers a positive environment for the control of the disease. It starts by tracing three phases in schistosomiasis control: disease elimination strategy through snail control (1950s-early 1980s); morbidity control strategy based on chemotherapy (mid 1980s to 2003); integrated control strategy (2004+). Each one of these phases took place in distinct policy-making environments. The paper partly draws on these phases to set out five issues of disease control and discusses them in the context of the health system and its recent trends. These cover the policy-making process, intersectoral action for health, equity and access to health services, funding for public goods and externalities, and strengthening resource management and planning. These issues form the basis of an agenda for integrating research and capacity strengthening in the Chinese health system with a view to creating a more positive enabling environment for schistosomiasis control. In so doing it is important to emphasize the role and integrity of the public sector against its commercialization, the underlying value of equity, a systems wide perspective, and the role of advocacy.

## Multilingual abstracts

Please see Additional file 1 for translations of the abstract into the six official working languages of the United Nations.

## Background

Schistosomiasis japonica is mainly prevalent in People’s Republic of China (P.R. China), the Philippines and small pockets of Indonesia, although P.R. China is the most heavily endemic of the three countries [1,2]. A large-scale epidemiological survey at the beginning of 1950s found that the disease was endemic in 10 provinces, one autonomous region and one municipality (city) mainly along the Yangtze River in the south of China [3]. It was estimated that 11.6 million people were infected with schistosomes and more than 100 million people were at risk of infection in the 1950s. There were 1.2 million infected cattle and the habitat area of *Oncomelania hupensis*, the intermediate host snails of *S. japonicum,* reached 14.3 billion m^2^. A great deal has been achieved in controlling schistosomiasis in P.R. China [1,4,5]. Between 1985 and 1995 disease transmission was interrupted in five provinces namely Guangdong, Shanghai, Fujian, Guangxi, and Zhejiang. By 2010, three provinces - Sichuan, Yunnan, and Jiangsu - reached the criteria of transmission control (both human and livestock prevalence less than 1%). Four other provinces characterized with complicated environments and easily affected by the water level of the Yangtze River- Hubei, Hunan, Jiangxi and Anhui - reached the criteria of infection control in 2008 (both human and livestock prevalence less than 5%). The number of infected cases has been reduced by over 97% since the 1950s, reaching the lowest historic level of 325,824 infections in 2010 [6]. The area of *Oncomelania hupensis* habitats was estimated to be 3.7 billion m^2^ which is about 25% of that in 1950s [7].

Having noted the above achievements, there are still many major challenges such as the existing extensive snail habitats with complicated environments, ecosystem changes caused by the construction of the Three Gorges Dams and the South–north Water Conversion Project, the effects of climate change, the scarcity of a highly sensitive surveillance and response system, and the access of infected persons to health care. This paper analyses the extent to which one of the keys to understand these challenges rests not only in the confines of the Schistosomiasis Diseases Control programme, but in the rest of the health system.

How diseases control programmes fit into health systems has been a recurrent theme of health systems analysis for many years. The debate over the vertical and/or horizontal nature of disease control programmes has occupied an important place in health systems analysis, together with discussions over the nature of integration and the specific circumstances in which integration is or is not appropriate [8-11]. Recent work has also looked to develop a more synergistic interrelationship between disease control programmes and the rest of the health system in addition to a more systems approach [12-15]. This paper comes within this line of analysis and focuses on a particular aspect; namely the extent to which the health system as a whole provides a positive environment for the effective development of disease control. It is based on a review of existing research, the analysis of which takes into account the research and practical experience of the authors.

Following this introduction, the paper identifies the historical phases in the control of schistosomiasis in P.R. China. The phases operated in specific political and social contexts and exhibited particular approaches to disease control. This leads to a consideration of an ‘enabling environment’ that we judge to significantly further disease control. On this basis, our analysis explores the extent to which the health system does or does not meet the needs of this ‘enabling environment’. Attention is paid to the policy-making process, intersectoral action for health, equity and access to health services, funding for public goods and services, and strengthening resource management and planning. Where appropriate, recommendations for an agenda of health systems research and development will be made. The paper concludes by analysing four emerging themes; the role and integrity of the public sector, the importance of equity for infectious diseases of poverty, the significance of health systems development, and the importance of advocacy. The paper is aimed at researchers, policy-makers and practitioners concerned with both schistosomiasis control and health systems development. It also suggests a line of analysis that can be developed in the analysis of other infectious diseases and their control, such as TB, malaria and HIV/AIDS.

### Schistosomiasis and its control in P.R. China

Schistosomiasis was one of the serious infectious diseases at the time of the founding of the P.R. China in 1949. Many famous terms, such as “Village without villagers” “Widows villages”, and “Big-belly village”, were used to describe the devastating consequences the disease brought to the Chinese people, especially the poor [3]. Since the 1950s China has been fighting the disease; strategies and approaches have evolved in a context of political, socio-economic, technological and epidemiological change. Three relatively distinct phases may be identified in the process of disease control: a) 1950s to early 1980s, b) mid-1980s to about 2003, c) from 2004 onwards. In identifying these phases we recognise a degree of generalisation in the analysis together with the overlapping nature of the phases. Each phase not only possesses an emphasis on certain approaches to disease control but relates to a policy environment of political and social change.

#### Disease elimination strategy with an emphasis on snails control (1950s to early 1980s)

Confronted by the poor state of health in P.R. China and with schistosomiasis as one of the main infectious diseases, there was strong political will among the leaders of the new republic to control the disease. However, the financial and human resources for healthcare in P.R. China were very limited, and most of them were distributed in a few urban cities. The infrastructure of China’s health systems in most places was poor and not up to the level of providing appropriate healthcare to the vast majority of the population. However, cooperation among different sectors was developed with programs receiving political support from a high level. In such circumstances, the Ministry of Health developed a policy of “Prevention First” in the 1950s and focused on snail control. Snail elimination with environmental modification and mollusciciding was emphasized in combination with chemotherapy. Mass movements were developed to mobilize community resources to contribute to the snail control campaign, through free labour, and local-driven innovative models for snail elimination. Under the auspices of the Communist Party, the Chinese people, as well as paramedics, such as barefoot doctors at the village level, engaged in disease control programmes. In the meantime, agricultural engineering and water conservancy activities, such as reclaiming wetlands, digging new ditches and filling the old ones, and changing rice paddies into dry crops were developed and implemented as a series of concerted actions to modify the snails’ habitats to be unsuitable for living and breeding.

An important feature of this disease control was the establishment and the operation of the vertical national schistosomiasis control programme in the 1950s. From national to provincial, prefectoral, county and township levels, anti-schistosomiasis institutions or stations were set up to take the main responsibility for disease control and treatment. The number of staff specialized in prevention and clinical care and working in these specialized organizations reached 17,000 by the mid 1960s, a powerful workforce fighting against the disease. The national programme, including these institutes and stations, was relatively well funded until the late 1970s. As results of the effective interventions, a large number of places in P.R. China were free of snails. The prevalence rate of schistosomiasis and new cases was reduced to a very low level in the early 1980s, particularly in the east coastal areas of China.

#### Morbidity control strategy based on chemotherapy (mid 1980s to about 2003)

China launched its economic reform in 1978, transforming its planned economy into a market one. The collective economy, based on the commune system in the rural areas, collapsed. A *de facto* privatization of agricultural production, named as “Household responsibility system” was introduced in 1983–84 in almost all the townships and villages. Chinese society has undergone profound changes since the economic reform. While many of these may be viewed as positive, such as improved living standards, there are important downsides, such as worsening equity and social justice. The diminished collective economy in the rural areas meant that the community-based health insurance schemes, called “Cooperative Medical Scheme”, collapsed in over 90% of townships and counties by the middle of the 1980s. Government health facilities received relatively less funding to cover their operational costs, while they were implicitly encouraged to increase service charges to support the provision of health services. The commercialization of healthcare has become widespread and common practice in health facilities in P.R. China while the health policy of “Prevention First” has to a large extent been neglected since the economic reform. Service providers became interested in generating revenues through service charges and profits from drug sale to cover costs and increase their incomes that were often linked to the level of revenue generation. Although the government still gave some support for control of schistosomiasis, but it was limited and could not meet the needs for disease control. Even the anti-schistosomiasis institutions and centres were required to generate revenues to cover partial costs of their operations. Few health facilities in P.R. China were still interested in engaging in preventive measures to control schistosomiasis, among other diseases. Furthermore, the mobilization of community resources for the disease control was no longer easy. Free labour was no longer available to tackle snail problems in rural communities, as the township and village leadership no longer had powers to force farmers to work on community projects for free. In addition, intersectoral action for health (IAH) became difficult, if not impossible. Market mechanisms have now come to dominate production in Chinese society, while the political push to promote intersectoral action for health and social development, which was strong in the planned economy, has been greatly weakened. By the late 1990s,some provinces and counties had scaled down the vertical programme and integrated many anti-schistosomiasis stations into general centre for disease control (CDC) systems or other disease prevention institutes.

By co-incidence, the WHO expert consultation committee for schistosomiasis control in 1984 adjusted its strategy and objectives for schistosomiasis control from transmission interruption or elimination to morbidity control in developing countries [16,17]. The new strategy focused on changing people’s behaviour with an objective to reduce the morbidity and mortality of schistosomiasis, rather than controlling the transmission of schistosomiasis completely; it was convinced that it would be formidably difficult to eliminate or interrupt the transmission of schistosomiasis without an enormous amount of financial investment in developing countries. With the support from the World Bank loan (1992–2001), the implementation of the strategy, emphasizing chemotherapy treatment of human being and livestock, as the main approach, was initiated in 1992 and completed in 2001. Different chemotherapy strategies were carried out in different endemic areas: mass chemotherapy was used for the people from endemic areas with a high prevalence and with a history of water contact. For residents and bovine in areas with medium endemicity, the treatment was only given to those with stool egg positive or positives in serological tests. In the area of low endemicity, only children were screened and treated, if diagnosed as positive cases. Infected cattle were also given appropriate treatments under the World Bank project [18]. The achievement of the World Bank funded project proved that the chemotherapy based strategy could decrease the prevalence of schistosomiasis quickly but that the consolidation task is arduous, as the areas of snail habitats are still large and fluctuated greatly in P.R. China [19-21]. Potential transmission remains considerable in the lake areas. In addition, the drugs for anti-schistosomiasis was free under the World Bank project, other costs of healthcare such as drugs for liver protection, were required to pay out of pocket by the patients. In the context of lack of appropriate health insurance schemes put in place, early case detection was often problematic. In a more general sense, access to healthcare in P.R. China was worsening in the 1990s, as seen in a rapid rise of healthcare costs, and increasing financial challenges the health insurance schemes in both urban and rural areas were facing. Following the completion of the World Bank project, the central government failed to come up with concrete policies supporting sustainable control of schistosomiasis, which leading to the resurgence of the schistosomiasis transmission after the World Bank loan project ended [22-24].

#### Integrated control strategy focusing on interrupting transmission (2004 –)

Since the late 1990s, the Government of P.R. China has increasingly recognized the important role the state should play in developing and strengthening health systems to improve equitable access to healthcare for the vast majority of the population. This responded to the increased inequity in health and healthcare and the resulting discontent among the public. The outbreak of SARS in 2003 was another alarm signal to the government that public health crises can affect not only health but also economic growth. Therefore, strengthening health systems to achieve universal health coverage has been put on the political agenda. A decision on the re-establishment of rural health insurance schemes with financial support from central government in 2002 was one of many examples that the government of P.R. China has once again taken health policies seriously.

Under these circumstances and with the re-emergence of schistosomiasis at the beginning of 21^st^ century in P.R. China, schistosomiasis control has once again been given a high priority. It was recognised that the chemotherapy-based approach could reduce the prevalence/morbidity to a low level rapidly. However, the environment of snail habitats would not change much and the opportunities of re-infection for many at-risk population remained high due to unchanged agricultural production and people’s life styles in endemic areas. Hence, a new integrated control strategy aimed to interrupt transmission based on reducing the rate of transmission of schistosomiasis infection from cattle and human being to snails has been developed and adopted by the national schistosomiasis control programme. The interventions include agricultural mechanization (to replace the use of cattle), supplying water, sanitation and lavatories/latrines, providing boats with faecal-matter containers, plus routine chemotherapy, moulluscides and health education [25,26]. These interventions have been made possible, owing to strong political, policy and financial support given to the national schistosomiasis control program in recent years. This forms part of the new health system reform in which increasing equitable access to public health interventions is one of top priorities set out by the government.

After several years implementation of the integrated control strategy, positive achievements have been seen. Four more provinces have now reached the level of infection control and three have met the targets of transmission control [6]. Compared to the situation in 2003, the number of estimated infected people reduced from 843,000 to 325,824. The number of acute cases decreased from 1,114 in 2003 to only 43 in 2010 [6]. The prevalence rate of infected cattle reduced from 4.1% to 1% over the period. Such results show that political will and appropriate policy on effective strategy on schistosomiasis control are critically important to effective disease control. Equally important is the strengthening of overall health systems from national to local level. Marketertization of healthcare, particularly public health programmes, would not work, especially in infectious disease control.

### Controlling schistosomiasis and the health system

This section will partly build on section 2 by drawing out and discussing five key issues of schistosomiasis control in P.R. China and how they relate to the health system. It will take into account the current trends in the health system and ask how these affect schistosomiasis control. It will also provide the grounds for recommending key areas of research and capacity development in the health system and in relation to schistosomiasis control.

Although a vertical form of schistosomiasis disease control programme was set in phase one (see section 2), there has been a process of integrating the activities of disease control with the CDC system and the general health services. A first area of research and policy analysis therefore is to analyse the extent of that integration, mapping out how this is expressed in resource generation and allocation, policy-making and planning, resource management, service delivery, and governance. This may be accompanied by an analysis of the determinants and impact of the diverse forms of integration in operation [27] and how the analysis of the five factors in this section would affect the degree of integration.

#### Policy-making

Section 2 raised important issues about policy-making for and the prioritising of schistosomiasis control in P.R. China. It was noted that different strategies of intervention have been used corresponding with the three phases which, in turn, corresponded with different policy-making environments. Although the first phase was characterised by depleted national resources, there was strong political will to control the disease; a robust vertical disease control programme was developed along with effective community involvement in transmission control, and intersectoral action for health. The second phase occurred in the context of market reform characterised by health care commercialisation and the loss of intersectoral action for health and community involvement. The phase coincided with a WHO supported shift to morbidity control through the World Bank supported project. The third phase constitutes a reaction to public health crises and the problems of inequity engendered through market reform and health care commercialisation.

The priority given to schistosomiasis control and the sustainability of interventions are certainly critical issues. These are important given the severity of the disease but also because it can easily rebound if attention is reduced. Section 2 emphasised the differences between the high priority given in the first and third periods and the lesser priority given in the second period. It goes without saying that policy-making operates in an historical context and the three phases clearly confirm this. Yet this raises a challenge to policy-making; how to achieve consistency and sustainability to undertake the medium to long term preventive interventions and intersectoral action for health as a form of disease control that goes beyond the shorter term political changes and the periodic form of loan financing. At the same time, the possibility of the disease to rebound requires a more sophisticated process of priority setting that relies primarily on the more immediate indicators of mortality and morbidity.

Prioritising schistosomiasis control within the broad range of health needs and interventions has to go beyond the spoken and written word of the policy declaration. Resource allocation has to give a material backing to the prioritising. Neither can this material backing be based on the assumption that regions and localities are able to raise their own funds for diseases control. This is particularly true for areas of central and western China which already receive central subsidies for rural health insurance, among others. Schistosomiasis is more prevalent in the poorer regions of China; Ross et al. noted that the disease “…remains a major problem in the marshland and lake areas of Hubei, Hunan, Anhui, and Jiangxi and in some mountainous areas of Sichuan and Yunnan” [28]. These are precisely among the regions of China that particularly require such subsidies and central funding.

Lastly, we need to emphasise that schistosomiasis control requires joint policy-making work across organisational boundaries and systems. Policy-making needs to move beyond the confines of the health system, a point we now pick up under the heading of intersectoral action for health.

#### Intersectoral action for health (IAH)

IAH is clearly important and comes through strongly as an important requirement of the health system for schistosomiasis control. The headlong imperative of economic growth can lead to growing inequality, poverty and disease burden. The precipitous implementation of water development projects and increased urbanisation has laid the conditions for increasing mortality and morbidity from schistosomiasis in P.R. China. To undercut the social and economic conditions of the disease requires a broad political perspective that puts the disease on the policy agenda, brings the disease control into the broader policy process identifying its social and economic conditions and secures the links between the disease control and health systems development. This was clearly more evident in the first and the third phases outlined in section 2. In the push for increased mechanisation in agriculture and improved water and sanitation, the health system needs to play its part in breaking the barriers within the health system and between the health and other systems such as agriculture, forestry and water / sanitation. It requires advocacy by actors involved with schistosomiasis control through the generation and presentation of evidence on the social economic conditions of schistosomiasis, policy analysis on the effectiveness and feasibility of interventions, together with networking and building coalitions of support for the control of the disease [29]. There is a need for this advocacy to be based on the underlying values of effective health interventions through IAH and equity.

An important constraint on IAH in P.R. China is the inward looking commercial practices of government sectors since the economic reform. Concerned with revenue growth and surplus generation, little is left for pooling resources and space for working together. While recent years have seen improvements in the central coordination of government actions – through the National Leading Group for Schistosomiasis Control and the Five Year Plan for Schistosomiasis Control – there is a need to monitor and evaluate the effectiveness of these organisational changes in leading to implementation of IAH at central, regional and local levels.

#### Equity and access to health services

Schistosomiasis is principally a disease of the poor and China is far from being an exception in this respect [22,30-32]. This raises a number of important issues: the extent to which there is an overall focus in the health system (and within schistosomiasis control) on equity and the poor, the social determinants of schistosomiasis and the impact the disease has on poverty, and the access to treatment for the poor. Those infected may use the Schistosomiasis Control Stations (SCS) although many of these have been integrated into the Centres of Disease Control (CDC). Treatment for schistosomiasis in these facilities is free at the point of delivery. However, before reaching these facilities, patients often pass through general health facilities (e.g. village health stations). There are certainly financial restrictions to access to these general health facilities for the poor, which can lead to further transmission of the disease mainly among the poor. The problem is that Yu et al’s study in 6 villages of Hunan Province in 2001 found “…both the willingness and the amount that people were willing to pay for treatment were low among villagers in the endemic areas in this region, especially in heavily endemic areas where villagers are most affected and have the lowest ability to pay” [33]. Although many are covered by the diverse forms of government sponsored health insurance in P.R. China, regulations on co-payments and ceilings negatively affect access. Linked to this are cases of rent seeking behaviour by providers such as supplier induced demand and mark-ups on medicines. Research into treatment of TB patients has also shown a certain lack of interest by general health care providers in referring patients on to the free care at the SCSs and CDCs, thus helping their own health care facility to reap the financial rewards [34]. Whether this occurs in the case of schistosomiasis needs to be the subject of research.

#### Funding for public goods and externalities

The control of schistosomiasis requires IAH, interventions that provide public goods and services together with goods with high externalities. This suggests the importance of appropriate and secure funding for disease control by the state and based on taxes or similar secure revenues [35]. These forms of disease control are not appropriate to financing and provision through private markets or commercialised public provision. The commercialisation of the health system in recent decades raises critical issues for schistosomiasis control in P.R. China. Restrictions and relative decreases in government budget allocations to health facilities, the increased dependence on user fees and insurance payments, the use of staff bonus schemes in health facilities based on treatment, the move from prevention to more revenue earning curative services, and the emergence of supplier induced demand raise serious doubts over the compatibility of schistosomiasis control and commercialisation of the public sector in P.R. China [35]. In theory, CDC and similar preventive institutions in P.R. China should be fully funded by the government. In reality, however, a vast majority of CDC need to raise some fund through service charges in order to cover partial operational costs and increase bonus payments to their staff. A key issue here is the need to develop general funding mechanisms based on tax bases and centrally or regionally allocated to affected regions to allow for the provision of public goods and preventive interventions required in the control of schistosomiasis.

#### Strengthening resource management and planning

Complementing the four previous factors of health systems development, there are a number of key areas in which the management and planning of resources need strengthening. These include surveillance and monitoring, human resource development for both specialist research and development in schistosomiasis control and the training of general health staff in the disease control, and the supply systems for delivering medicines. Monitoring the effectiveness of implementing schistosomiasis control is a key challenge. There are two important areas for development: firstly, there is the need to ensure targets met with the delivery of high quality services, and secondly, to ensure efficiency in resource utilisation. Another most important issue is how to improve positive synergies of combining the local resources with the fund from the central government. Some interventions, such as mollusciciding, chemotherapy of local residents and bovines, and faecal management need appropriate resource pooling to increase the population coverage, while some engineering related interventions, such as agriculture irrigation system modification, altering the crops planting, biogas station should pool resources from different channels in an effective way. The high cost-effectiveness for a specific strategy in the either vertical or integrated control program relies on good resource management. In the current status of P.R. China, it is essential for the national control programme in P.R. China to be part of the push for universal coverage of health care in order to ensure sustainable control of schistosomiasis in P.R. China.

### Moving forward to health systems development

The five issues featured in the previous section constitute an agenda for integrated research and capacity strengthening in health systems with a view to schistosomiasis control. Applied research should increase our understanding of the health system needs of schistosomiasis control, while development strengthens the capacity of the health system to meet the needs of disease control. In so doing, there are four important considerations.

a) An important theme running through this paper is to shift away from the commercialisation of the public sector and move towards strengthening of the role and integrity of the public sector in schistosomiasis disease control. This is apparent in the strengthening of policy-making in government, intersectoral action for health, the importance of equity as a key value, the access to treatment, the provision of public goods and the strengthening of resource management and planning. These need to be broken down into specific measures, such as strengthening surveillance systems, urban and rural health insurance, and funding for public goods and services.

b) An underlying value of the whole approach to the control of schistosomiasis is that of equity. On the one hand, this requires a reaffirmation of public service values around health systems based on improved and more equitable health and health care. On the other, it is a disease of poverty, the control of which needs to get to the foundations of that poverty.

c) The call for research and development in these five areas should not lead to isolation of specific forms of disease control. Many of these features of disease control hold for other diseases. At the same time, care needs to be taken in dealing with these issues in a system wide perspective. For example, setting the priority of schistosomiasis control in policy-making and resource control needs to be seen in the context of general health needs and general health planning. The exercise of health needs identification needs to be done across the full spectrum of infectious disease of poverty and, in fact, the overall health needs of a society. The exercise conducted in this paper needs to be one of many exercises in disease control and in which health systems change is responding to health needs of a population.

d) Lastly, we return to advocacy. It is anticipated that the discussion of this and other likeminded papers should lead to ‘a health systems agenda for schistosomiasis control’. Such an agenda needs to coalesce around a coalition of stakeholders; those support of sufferers of the disease, researchers from diverse disciplines, technical experts, politicians, health managers and planners, and service providers.

## Abbreviations

CDC: Centre for disease control; IAH: Intersectoral action for health; SARS: Severe acute respiratory syndrome; SCS: Schistosomiasis control stations; TB: Tuberculosis.

## Competing interests

The authors have no competing interests.

## Authors’ contributions

CC, the honorary reader at Liverpool School of Tropical Medicine, participated in the initial design of the paper and in its writing, JX did the literature review used to develop the paper and also participated in the writing, and ST participated in the initial design of the paper with CC, and drafted some sections of the paper. All authors reviewed and approved the final version.

## Supplementary Material

Additional file 1 Multilingual abstracts in the six official working languages of the United Nations. Click here for file

## References

[B1] Chitsulo L Engels D Montresor A Savioli L The global status of schistosomiasis and its control Acta Trop 2000 77 1 41 51 10.1016/S0001-706X(00)00122-4 10996119 PMC5633072

[B2] Gryseels B Polman K Clerinx J Kestens L Human schistosomiasis Lancet 2006 368 9541 1106 1118 10.1016/S0140-6736(06)69440-3 16997665

[B3] Chen MG Feng Z Schistosomiasis control in China Parasitol Int 1999 48 1 11 19 10.1016/S1383-5769(99)00004-5 11269321

[B4] Engels D Chitsulo L Montresor A Savioli L The global epidemiological situation of schistosomiasis and new approaches to control and research Acta Trop 2002 82 2 139 146 10.1016/S0001-706X(02)00045-1 12020886 PMC5633073

[B5] Wang LD Utzinger J Zhou XN Schistosomiasis control: experiences and lessons from China Lancet 2008 372 9652 1793 1795 10.1016/S0140-6736(08)61358-6 18930529 PMC7135384

[B6] Lei ZL Zheng H Zhang LJ Zhu R Guo JG Wang LY Chen Z Zhou XN Schistosomiasis endemic status in People's Republic of China in 2010 Chin J Schisto Control 2011 23 6 599 604 in Chinese 22379811

[B7] Zhen J A brief introduction to a nation-wide sampling survey on schistosomiasis Chin Med J (Engl) 1993 106 8 569 575 8222904

[B8] Mills A Vertical vs horizontal health programmes in Africa: idealism, pragmatism, resources and efficiency Soc Sci Med 1983 17 24 1971 1981 10.1016/0277-9536(83)90137-5 6670002

[B9] Cruz VO Kurowski C Mills A Delivery of priority health services: searching for synergies within the vertical and versus horizontal debate J Int Develop 2003 15 67 86 10.1002/jid.966

[B10] Unger JP De Paepe P Green A A code of best practice for disease control programmes to avoid damaging health care services in developing countries Int J Health Plann Manage 2003 18 Suppl 1 S27 39 14661939 10.1002/hpm.723

[B11] Ooms G Van Damme W Baker BK Zeitz P Schrecker T The 'diagonal' approach to Global Fund financing: a cure for the broader malaise of health systems? Global Health 2008 4 6 10.1186/1744-8603-4-6 18364048 PMC2335098

[B12] WHO Report on the 2nd Expert Consultation on Positive Synergies between Health Systems and Global Health Initiatives, Mexico City, Mexico, 5 August 2008 Maximising Positive Synergies between Health Systems and Global Health Initiatives 2008 WHO, Geneva

[B13] WHO Report on the Expert Consultation on Positive Synergies between Health Systems and Global Health Initiatives Maximising Positive Synergies between Health Systems and Global Health Initiatives 2008 Geneva: WHO

[B14] Collins C Gonzalez Block MA Tang S Disease control and health systems in low- and middle-income countries: enhancing positive interrelation Trop Med Int Health 2012 17 5 646 651 10.1111/j.1365-3156.2012.02968.x 22420372

[B15] WHO Systems Thinking for Health Systems Strengthening Geneva, Switzerland: World Health Organization http://www.who.int/alliance-hpsr/resources/9789241563895/en/index.html

[B16] WHO World health organization new strategy on schistosomiasis control Southeast Asian J Trop Med Public Health 1984 15 469 470 6535260

[B17] WHO The control of schistosomiasis WHO Technical report series 1993 Geneva: WHO 830

[B18] Chen XY Wang LY Jiming C Zhou XN Zheng J Guo JG Wu XH Dirk E Chen MG Schistosomiasis control in China: the impact of a 10-year World Bank Loan Project (1992–2001) Bull World Health Organ 2005 83 1 43 48 15682248 PMC2623468

[B19] Wang RB Wang TP Wang LY Guo JG Yu Q Xu J Gao FH Yin ZC Zhou XN Study on the re-emerging situation of schistosomiasis epidemics in areas already under control and interruption Chin J Epidemiol 2004 25 7 564 567 in Chinese 15308033

[B20] Wu XH Xu J Zhou XN Guo JG Challenges and control strategies in areas of schistosomiasis transmission controlled and interrupted in China Chin J Schisto Control 2004 16 1) 1 3 in Chinese

[B21] Zhou XN Wang LY Chen MG Wu XH Jiang QW Chen XY Zheng J Utzinger J The public health significance and control of schistosomiasis in China–then and now Acta Trop 2005 96 2–3 97 105 16125655 10.1016/j.actatropica.2005.07.005

[B22] Zhou XN Guo JG Wu XH Jiang QW Zheng J Dang H Wang XH Xu J Zhu HQ Wu GL Epidemiology of schistosomiasis in the People's Republic of China, 2004 Emerg Infect Dis 2007 13 10 1470 1476 10.3201/eid1310.061423 18257989 PMC2851518

[B23] Zhou XN Bergquist R Leonardo L Yang GJ Yang K Sudomo M Olveda R Schistosomiasis japonica control and research needs Adv Parasitol 2010 72 145 178 20624531 10.1016/S0065-308X(10)72006-6

[B24] Liang S Yang C Zhong B Qiu D Re-emerging schistosomiasis in hilly and mountainous areas of Sichuan, China Bull World Health Organ 2006 84 2 139 144 10.2471/BLT.05.025031 16501732 PMC2626530

[B25] Wang LD Guo JG Wu XH Chen HG Wang TP Zhu SP Zhang ZH Steinmann P Yang GJ Wang SP China's new strategy to block Schistosoma japonicum transmission: experiences and impact beyond schistosomiasis Trop Med Int Health 2009 14 12 1475 1483 10.1111/j.1365-3156.2009.02403.x 19793080

[B26] Wang LD Chen HG Guo JG Zeng XJ Hong XL Xiong JJ Wu XH Wang XH Wang LY Xia G A strategy to control transmission of Schistosoma japonicum in China N Engl J Med 2009 360 2 121 128 10.1056/NEJMoa0800135 19129526

[B27] Atun R de Jongh T Secci F Ohiri K Adeyi O Integration of targeted health interventions into health systems: a conceptual framework for analysis Health Policy Plan 2010 25 2 104 111 10.1093/heapol/czp055 19917651

[B28] Ross AG Sleigh AC Li Y Davis GM Williams GM Jiang Z Feng Z McManus DP Schistosomiasis in the People's Republic of China: prospects and challenges for the 21st century Clin Microbiol Rev 2001 14 2 270 295 10.1128/CMR.14.2.270-295.2001 11292639 PMC88974

[B29] Collins C Green A Valuing Health Systems: A Framework for Low and Middle Income Countries 2012 New Delhi: Sage

[B30] Vandemark LM Jia TW Zhou XN Social science implications for control of helminth infections in Southeast Asia Adv Parasitol 2010 73 137 170 20627142 10.1016/S0065-308X(10)73006-2

[B31] Zhou XN Lv S Yang GJ Kristensen TK Bergquist NR Utzinger J Malone JB Spatial epidemiology in zoonotic parasitic diseases: insights gained at the 1st International Symposium on Geospatial Health in Lijiang, China, 2007 Parasit Vectors 2009 2 10 10.1186/1756-3305-2-10 19193214 PMC2663554

[B32] Zhou XN Prioritizing Research for "One health-One world" Inf Dis Poverty 2012 1 1 1 10.1186/2049-9957-1-1 PMC3710101 23849840

[B33] Yu DB Manderson L Yuan LP Wei WY He HB Chen Y Is equity being sacrificed? Willingness and ability to pay for schistosomiasis control in China Health Policy Plan 2001 16 3 292 301 10.1093/heapol/16.3.292 11527870

[B34] Liu X Thomson R Y G Zhao F Squire SB Tolhurst R Zhao X Yan F Tang S How affordable are tuberculosis diagnosis and treatment in rural China? An analysis from community and tuberculosis patient perspectives Trop Med Int Health 2007 12 12 1464 1471 10.1111/j.1365-3156.2007.01953.x 18076553

[B35] Bian Y Sun Q Zhao Z Blas E Market reform: a challenge to public health–the case of schistosomiasis control in China Int J Health Plann Manage 2004 19 Suppl 1 S79 94 15686062 10.1002/hpm.771

