# Supplementary material for: Schistosomiasis control and the health system in P.R. China
Source: Infect Dis Poverty. 2012 Nov 1;1:8. doi: 10.1186/2049-9957-1-8 (PMC3710143; doi:10.1186/2049-9957-1-8)
Supplement: Additional file 1 — Multilingual abstracts in the six official working languages of the United Nations. [file 2049-9957-1-8-S1.pdf]

Translation of the abstract into the six official working languages of the United Nations

مكافحة البلهارسيا والنظام الصحي في الصين

تشارلز كولينز ، جينغ شو، و شينجلان تانغ.

خلاصة

على مدى السنوات الستين الماضية تم احراز تقدم في مكافحة مرض البلهارسيا في الصين. و مع ذلك لا تزال التحديات صعبة . تنظر هذه المقالة في مدى توفيرالنظام الصحي للبيئة الإيجابية للسيطرة على المرض . وتبدأ من خلال تتبع ثلاث مراحل في مكافحة داء البلهارسيا:استراتيجية القضاء على المرض عن طريق التحكم في الحلزون (الخمسينات-أوائل الثمانينات)؛ استراتيجية مكافحة الاعتلال استناداً إلى العلاج الكيميائي (منتصف الثمانينات إلى عام 2003)؛ و استراتيجية المكافحة المتكاملة (2004++). و جرت كل واحدة من هذه المراحل في بيئات متميزة في صنع السياسات. و تعتمد المقالة، الى حد ما، على هذه المراحل لوضع خمسة مسائل للتحكم بالمرض و مناقشتهم في اطار النظام الصحي و التوجهات الجديدة. وتغطي هاته المسائل عملية وضع سياسة العمل ، العمل المشترك بين قطاعات الصحة ، الإنصاف في الوصول إلى الخدمات الصحية ، تمويل السلع العمومية والعوامل الخارجية، وتعزيز إدارة الموارد والتخطيط . هذه القضايا تشكل الأساس لجدولة أعمال الإدماج في البحوث و تعزيز قدرات النظام الصحي الصيني بغية تهيئة بيئة مواتية و أكثر إيجابية لمكافحة البلهارسيا. و من المهم أثناء القيام بذلك التأكيد على دور و نزاهة القطاع العام في تسويقها ، مفهوم العدالة الصحية كقيمة أساسية ، النظرة الشاملة للأنظمة ، و دور الدعم .

Translated from English version into Arabic by Nesrine Echroudi, through

TRANSLATORS
WITHOUT BORDERS

中国的血吸虫病控制及其卫生体制

Charles Collins, Jing Xu and Shenglan Tang

摘要

在过去的 60 多年，中国血吸虫病防治取得了很大的成就，但仍面临挑战。本文着眼于卫生体制能在多大程度上为该病控制提供积极环境。首先回顾了血吸虫病控制的 3 个阶段：控制钉螺以消除疾病的策略（20 世纪 50 年代至 80 年代早期）、化疗基础上的发病控制策略（20 世纪 80 年代中期至 2003 年）、综合防治策略（2004 年至今）。每个阶段都发生在独特的政策制定环境下。本文从这些阶段提取信息陈列了疾病控制的 5 个论点，并在卫生体制的背景下讨论这些要点及其新近的趋势，包括政策制定过程、部门间合作、卫生服务的公正性和可及性、公共财产及其外部性的资助、加强资源管理和规划等。这些论点形成了在中国卫生体制中整合研究和加强能力的议程基础，以为血吸虫病控制提供更加积极的环境。在这种情况下，强调公共部门的作用和完善以反对其商业化、公正的潜在价值、体制的宽视野以及宣传倡导的作用是十分重要的。

Translated from English version into Chinese by Xu Jing

Contrôle de la bilharziose ou schistosomiase et système de santé en Chine

Charles Collins, Jing Xu et Shenglan Tang

Extrait

Ces soixante dernières années, des progrès ont été faits dans le contrôle de la bilharziose en Chine. Il reste cependant des défis difficiles à relever. Cet article porte sur la mesure dans laquelle le système de santé offre un environnement positif de contrôle de la maladie. Il commence par décrire les trois phases du contrôle de la bilharziose : stratégie d'élimination de la maladie par le contrôle des escargots (des années 1950 aux années 1980) ; stratégie de contrôle de la morbidité fondée sur la chimiothérapie (milieu des années 1980 à 2003) ; stratégie de contrôle intégrée (2004 et après). Chacune de ces phases a eu lieu dans des environnements décisionnels bien distincts. L'article se base en partie sur ces différentes phases pour définir cinq problèmes de contrôle de la maladie et en débat dans le contexte du système de santé actuel et de ses récentes tendances. Celles-ci couvrent le processus de décision, l'action intersectorielle pour la santé, l'équité et l'accès aux services de santé, le financement de marchandises publiques et externalités et le renforcement de la gestion et de la planification des ressources. Ces questions forment la base d'un programme d'intégration de la recherche et du renforcement de la capacité dans le système de santé chinois, en vue de créer un environnement favorable plus positif pour le contrôle de la bilharziose. Ce faisant, il est important de souligner le rôle et l'intégrité du secteur public par rapport à sa commercialisation, la valeur sous-jacente de l'équité, une perspective globale des systèmes et le rôle à jouer par la sensibilisation.

Translated from English version into French by Delphine LE BOULANGER-REID, through

TRANSLATORS
WITHOUT BORDERS

Контроль шистосомоза и система здравоохранения в Китае

Чарльз Коллинс, Джинг Ксу и Шенглен Тенг

Аннотация

За последние шестьдесят лет сделаны достижения в контроле шистосомоза в Китае. Тем не менее, все еще существуют сложные проблемы для решения. Данная работа широко рассматривает, какая из систем здравоохранения предоставляет благоприятную среду для контроля заболевания. Она начинается прослеживанием трёх фаз контроля шистосомоза: стратегия устранения заболевания через контроль шистосомоза (1950-е - начало 1980-х); стратегия контроля частоты заболеваний, основанной на химиотерапии (середина 1980-х до 2003 г.); стратегия комплексного контроля (2044+). Каждая из этих фаз совершается в определённой разработке политики среды. Работа частично основана на этих фазах, чтобы представить пять целей контроля заболевания, и обсудить их в контексте системы здравоохранения и ее последних направлений. Они охватывают разработку политики процесса, межсекторную работу для здравоохранения, объективность и доступ к услугам здравоохранения, финансирование общественных благ и экзогенных факторов, а также укрепление управления и планирования ресурсов. Данные цели формируют основу программы для интегрированного изучения и способности укрепления китайской системы здравоохранения с целью создания положительной благоприятной среды для контроля шистосомоза. При этом важно подчеркнуть роль и целостность государственного сектора против его коммерциализации, основных ценностей объективности, системы широких перспектив, и роли защиты.

Translated from English version into Russian by Halyna Maksymiv, through

TRANSLATORS
WITHOUT BORDERS

Control de la esquistosomiasis y el sistema sanitario en China

Charles Collins, Jing Xu y Shenglan Tang

Resumen

En los últimos sesenta años se ha avanzado en el control de la esquistosomiasis en China. Existen, sin embargo, aún quedan retos difíciles a los que enfrentarse. Este papel examina hasta qué punto el sistema sanitaria proporciona un entorno positivo para el control de esta enfermedad. Empieza por seguir las tres fases en el control de la esquistosomiasis: estrategia de eliminación de la enfermedad a través del control de los caracoles (años 50 a principios de los 80); estrategia de control de la morbilidad basada en la quimioterapia (mediados de los años 80 al 2003); estrategia integrada de control (a partir del 2004). Cada una de estas fases se realizó en entornos decisorios distintos. El papel utiliza parcialmente estas fases para establecer cinco cuestiones del control de la enfermedad y las analiza en el contexto del sistema sanitario y sus últimas tendencias. Estas abarcan el proceso de toma de decisiones políticas, la acción intersectorial para la salud, equidad y acceso a los servicios sanitarios, financiación de los bienes públicos y externalidades, y el fortalecimiento de la gestión y planificación de recursos. Estas cuestiones forman la base de una agenda para integrar la investigación y el fortalecimiento de la capacidad en el sistema sanitario chino con la vista puesta en la creación de un entorno más positivo que permita el control de la esquistosomiasis. Al hacerlo así, es importante enfatizar el papel y la integridad del sector público contra su comercialización, el valor subyacente de la equidad, una perspectiva amplia del sistema y el papel de la abogacía.

Translated from English version into Spanish by Lizette Britz, through

TRANSLATORS
WITHOUT BORDERS
